# Temperature Characteristics Modeling for GaN PA Based on PSO-ELM

**DOI:** 10.3390/mi15081008

**Published:** 2024-08-05

**Authors:** Qian Lin, Meiqian Wang

**Affiliations:** 1 School of Intelligent Science and Engineering, Qinghai Minzu University, Xining 810007, China; 17697249359@139.com; 2 School of Electronic Science and Engineering, University of Electronic Science and Technology, Chengdu 610050, China; 3 Tong Fang Electronic Technology Company, Jiujiang 332000, China

**Keywords:** GaN PA, PSO-ELM, ELM, temperature characteristic, modeling

## Abstract

In order to solve the performance prediction and design optimization of power amplifiers (PAs), the performance parameters of Gallium Nitride high-electron-mobility transistor (GaN HEMT) PAs at different temperatures are modeled based on the particle swarm optimization–extreme learning machine (PSO-ELM) and extreme learning machine (ELM) in this paper. Then, it can be seen that the prediction accuracy of the PSO-ELM model is superior to that of ELM with a minimum mean square error (MSE) of 0.0006, which indicates the PSO-ELM model has a stronger generalization ability when dealing with the nonlinear relationship between temperature and PA performance. Therefore, this investigation can provide vital theoretical support for the performance optimization of PA design.

## 1. Introduction

Today, with the rapid development of wireless communication technology, power amplifiers (PAs) play an important role in the modern wireless communication system. Therefore, the performances of PA have a decisive influence on the transmission quality and the efficiency of the whole system [1,2]. Meanwhile, the requirements for the stability and reliability of PAs on various occasions are increasingly urgent [3,4]. Therefore, it is of great significance to investigate the nonlinear relationship between PA performance and temperature.

In previous studies, it can be found that some degree of performance degradation was caused by temperature [5,6,7,8,9,10]. Several scholars have investigated the nonlinear relationship between PA performance and temperature. For example, in 2018, Zhou proposed a neural network modeling method for PA based on temperature effects [11]. In 2021, Jha studied the behavior modeling of Gallium Nitride (GaN) class AB dual-frequency PA at different temperatures and humidities and emphasized the importance of the environment for PAs [12]. In 2022, a Support Vector Machine (SVM) was utilized to model a Complementary Metal Oxide Semiconductor (CMOS) PA [13]. In the same year, S-parameters of PAs at different temperatures were modeled based on the BP neural networks by Zhao [14]. Meanwhile, Lin discussed the application of the X parameter in the modeling of microwave power devices [15]. Yang modeled the key indicators of RF amplifiers using an extreme learning machine (ELM) and verified the accuracy of the model in 2023 [16]. In 2024, Lin conducted behavioral modeling for a Gallium Arsenide (GaAs) pseudomorphic high-electron-mobility transistor (pHEMT) monolithic microwave, integrating circuit (MMIC) high-gain PAs at different temperatures [17]. However, how to build models with more generalization ability to predict PA performance accurately on complex occasions is still the current challenge. In recent years, ELM has emerged as a novel machine learning algorithm with continuous advancement, and it has shown significant advantages in many fields with rapid and efficient learning capabilities. It can effectively deal with complex nonlinear relationships and accurately capture the impact of factors such as temperature.

However, ELM is limited by the number of hidden layer nodes and parameters. In order to further optimize the performance of ELM, PSO and ELM are combined to achieve the global optimization of hidden layer nodes and improve the accuracy of modeling. Therefore, a GaN PA is used as the modeling object; the performance parameters were predicted based on PSO-ELM and ELM at −40 °C, 0 °C, 40 °C, 80 °C, and 120 °C, respectively. The prediction results show that PSO-ELM has a higher accuracy than ELM with a minimum mean square error (MSE) of 0.0006.

The paper is organized as follows: the modeling object and methods are explained in Section 2, as well as the detailed modeling procedure. In Section 3, the modeling results are discussed. Finally, concluding remarks are presented in Section 4.

## 2. Modeling Object and Methods

### 2.1. Modeling Object

**Figure 1:**
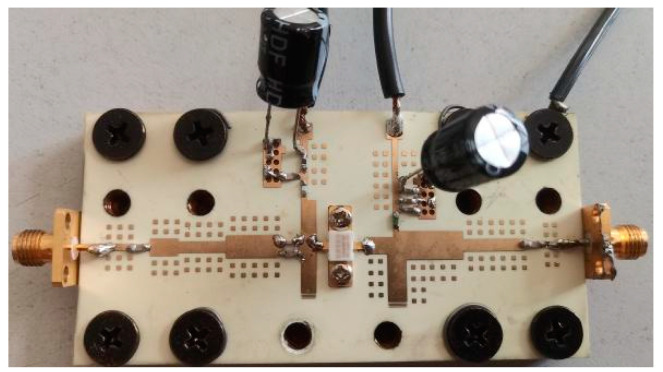
Physical photograph of the GaN PA.

In this paper, a broadband high-efficiency GaN PA is chosen as the object. The step-matching structure and harmonic control network are used to control the second and third harmonics in this PA, which can greatly improve its efficiency and bandwidth. The physical photograph of the PA is shown in Figure 1.

After the design and simulation in ADS, a series of temperature simulations were carried out to obtain the S-parameters and large signals of the PA at different temperatures.

### 2.2. Modeling Methods

#### 2.2.1. ELM

**Figure 2:**
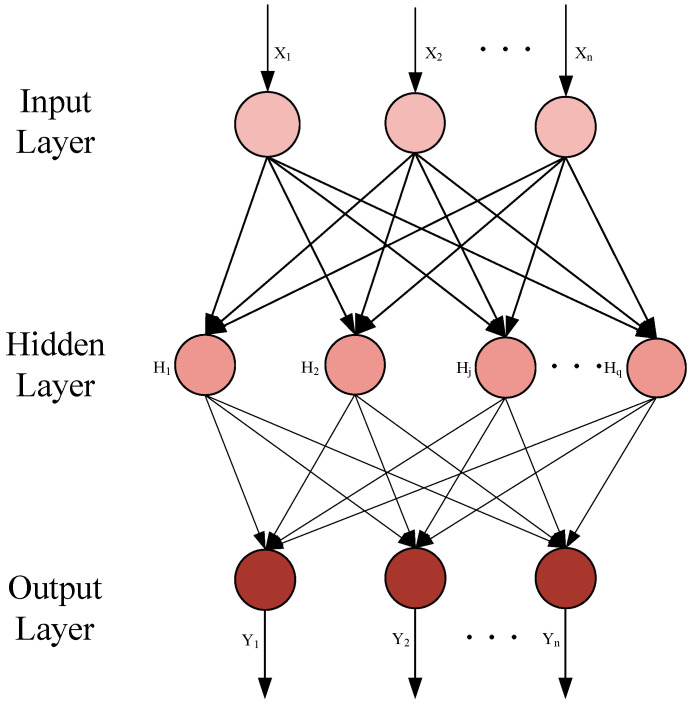
Basic structure of ELM for GaN PA.

ELM was proposed by Huang in 2004 [18], which is a feed-forward neural network (FNN) with a single hidden layer. It has the advantages of simple training, strong generalization ability, and fast training. Therefore, it is widely used in pattern recognition [19], regression analysis [20], classification [21], and other fields. The basic structure of ELM is described in Figure 2, which is composed of the input layer, hidden layer, output layer, and the neurons for each layer. The number of neurons for the input layer and output layer is determined by the input vector of X = [X_1_, X_2_, …, X_n_] and the output vector of Y = [Y_1_, Y_2_, …, Y_n_]. Then, the neuron number of hidden layers can be adjusted according to the complexity of the nonlinear problem. During training, the input weight matrix and hidden layer threshold matrix can be randomly selected; thus, the solution can be solved by adjusting the number of neurons in the hidden layer [22].

There are three steps for ELM training, detailed as follows:

Firstly, it is necessary to determine the number of neurons of L and j for the input layer and the hidden layer. Meanwhile, the input vector of X, the weight of w, and the bias of b also should be given according to the nonlinear function.

Then, after selecting the activation function, the output matrix of H in the hidden layer can be calculated by Formula (1):

(1) $$H=\sum_{j=1}^{L}\beta_{j}g(\omega X+b)$$

where g is a nonlinear activation function that is needed for nonlinear classification, and it is used to approximate the nonlinear target function.

Finally, for the output sample of T, the output weight matrix can be obtained by solving the least square. Thus, the estimated value $\hat{\beta }$ is shown as Equation (2):

(2) $$\hat{\beta }=H^{+}T$$

where $H^{+}$ is the generalized inverse matrix of the hidden layer output function H.

Through the above process, ELM is trained and tested for several cycles to generate an ELM prediction model until the accuracy is good.

#### 2.2.2. PSO-ELM

PSO was first proposed by Kennedy and Eberhart in 1995 [23], which is a kind of intelligent optimization algorithm that can simulate the foraging behavior of birds. Its basic idea is to find the optimal solution through cooperation and information sharing among individuals. In order to optimize the modeling, PSO-ELM is generated with PSO and ELM, which can calculate the fitness of each particle through particle swarm operation and adjust the speed and position of particles to obtain the optimal weight and threshold constantly. Thus, the global optimal solution can be obtained through group cooperation until the preset network termination conditions can be met. Finally, the prediction results with high efficiency and accuracy can be achieved.

The update velocity of each particle is shown in Equation (3):

(3) $$\omega V_{i}^{k}+c_{1}r_{1}(P_{i}^{k}-X_{i}^{k})+c_{2}r_{2}(P_{g}^{k}-X_{i}^{k})$$

The position update of each particle is shown in Equation (4):

(4) $$X_{i}^{k+1}=X_{i}^{k}+V_{i}^{k+1}$$

where k is the number of iterations; $V_{i}^{k}$ is the particle velocity; $P_{i}^{k}$ is the optimal position of the current particle, which is the current particle can be found from generation 0 to generation k; $X_{i}^{k}$ is the particle position; $P_{g}^{k}$ is the population optimal position; c_1_ and c_2_ are the learning factors, which can affect the convergence of the particle; r_1_ and r_2_ are the random numbers; and ω is the inertia weight.

In general, it is repeated until it converges.

### 2.3. Modeling Process

#### 2.3.1. Modeling of ELM

**Figure 3:**
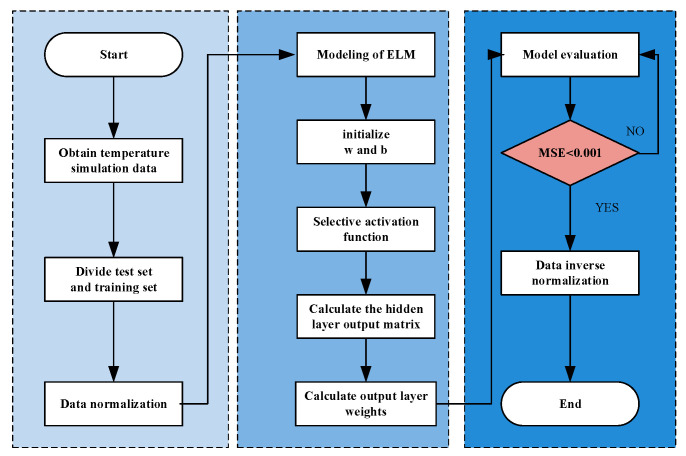
Modeling flowchart of ELM model for GaN PA.

According to the above analysis, ELM can better model the nonlinear relationship between temperature and PA performance. The modeling flowchart of ELM for this GaN PA is shown in Figure 3.

It can be seen that the major process for the ELM model is as follows:

Step 1: The simulation data of the PA at different temperatures are divided into training data and testing data.

Step 2: Data normalization. Here, the activation function is used to normalize the input and output of the training set.

Step 3: ELM model construction.

Step 4: Sigmoid is selected as the activation function.

Step 5: Calculate the hidden layer output matrix. Using randomly generated weights and biases, the output matrix of the training set and testing set can be calculated.

Step 6: Calculate the weight of the output layer. The least square method is used to calculate the optimal weight from the hidden layer to the output layer.

Step 7: Model evaluation. Use the testing sets to evaluate the performance of the model.

Step 8: When comparing the testing set with the predicted results of ELM, if the error is larger than 0.001, the number of neurons and weights will be adjusted. When the error is less than 0.001, the prediction models are generated. The mean square error is picked as an index for determining the accuracy of the model here. Its size can reflect the deviation of the predicted from the true value, and it can be obtained from Equation (5) [24]:

(5) $$MSE=\frac{1}{n}\sum_{i=1}^{n}{\left(P_{i}-T_{i}\right)}^{2}$$

where MSE is the result of the mean square error, n represents the number of prediction samples, P_i_ is the i_th_ predicted value, T_i_ is the corresponding i_th_ expected value, and $\bar{P_{i}}$ is the mean of the i_th_ predicted value.

In addition, in order to evaluate the accuracy of the model, Nash-Sutcliffe Efficiency (NSE) is also calculated, which can be calculated from Equation (6) [25]:

(6) $$NSE=1-\frac{\sum_{i-1}^{n}{\left(P_{i}-T_{i}\right)}^{2}}{\sum_{i-1}^{n}{\left(P_{i}-\bar{P_{i}}\right)}^{2}}$$

Step 9: Generate the prediction model. If the error can meet the accuracy, the prediction model is generated and the modeling process is ended.

#### 2.3.2. Modeling of PSO-ELM

**Figure 4:**
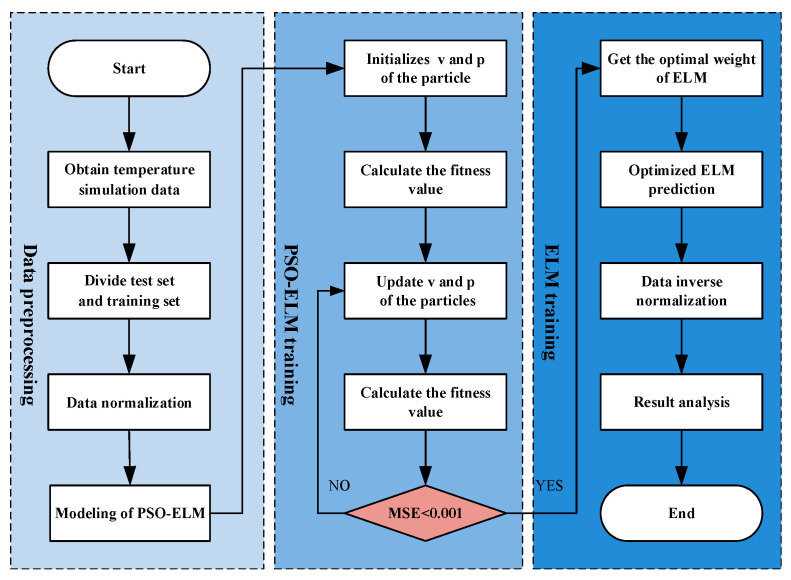
Modeling flowchart of PSO-ELM for GaN PA performance.

The random generation of w and b in the initialization of ELM is unstable and will affect the prediction accuracy [26]. Therefore, PSO is introduced to optimize the values of w and b of ELM. The modeling flowchart of PSO-ELM is shown in Figure 4.

This process consists of four steps, detailed as follows:

Step 1: Data acquisition. T and F are the independent variables and Pin, S_21_, S_11_, S_22_, Pout, and Gain are the dependent variables.

Step 2: Training and testing sets are divided.

Step 3: Data normalization.

Step 4: PSO-ELM model construction.

Step 5: Initialization of PSO algorithm. Here, the number of nodes in the hidden layer is 8, the population size is 25, the maximum number of particle iterations is 150, the learning factor is 1.5, the maximum inertia weight is 0.9, and the minimum inertia weight is 0.4. Meanwhile, the position of the particle is at [0, 1], the activation function of the hidden layer is sigmoid, the maximum number of ELM is 1000, the learning rate is 0.0001, and the error accuracy is 0.0004.

Step 6: Calculate the initial fitness value and find out the individual and global optimal position.

Step 7: Iteration of PSO algorithm. The updated fit values are calculated, and the individual and global optimal positions are updated.

Step 8: Update particle position and velocity. The position and velocity of the particle are updated according to the current position and velocity and the information of the individual and global optimal position.

Step 9: ELM training and prediction. The testing set is trained and predicted with ELM; meanwhile, the training data and testing data are fitted and the predicted results are de-normalized.

Step 10: Result analysis.

Here, the mean square error is also selected as the major index to judge the accuracy of the model, and NSE is also used to evaluate the accuracy.

In addition, the performance and generalization ability of models can be affected by the hyperparameters of machine learning [23]. Therefore, in order to optimize the complexity, the learning ability, and the generalization ability of the model and improve the prediction accuracy, it is necessary to adjust its hyperparameters.

The first hyperparameter is the number of hidden layer nodes, which can affect the complexity and learning ability of the model directly. The number of hidden layer neurons of PSO-ELM and ELM is given as Formula (7):

(7) $$S=\sqrt{m+n}+\delta$$

where S is the number of neurons in the hidden layer, n is the number of neurons in the input layer, m is the output layer, and δ is a constant between 1 and 10.

To determine the best number of hidden layer neurons, experiments should be conducted according to the empirical formula. Furthermore, the average MSE of ten times is used to measure the number of hidden layer neurons. When the number of hidden layer neurons in PSO-ELM and ELM is 12, the MSE is the smallest. Therefore, the optimal number of hidden layer neurons for two models can be determined.

The second hyperparameter is the activation function. The sigmoid and relu are the most widely used in an artificial neural network. Here, the modeling effects of these two activation functions are compared and sigmoid is selected.

The third hyperparameter is the learning rate. The step size of parameter updating is determined. Therefore, the appropriate learning rate can make the objective function converge to the global minimum in a reasonable time. In this paper, the learning rate of PSO-ELM and ELM is set to 0.0001 and 0.001, respectively.

The fourth hyperparameter is the number of iterations. The number of rounds is determined. Therefore, in this paper, the iterative times of PSO-ELM and ELM are set to 150 and 1000, respectively.

In addition, the particle number, inertia weight, and learning factor of PSO are also important hyperparameters. Therefore, in this paper, the population size of PSO-ELM is 25, the learning factors are 1.5, the maximum inertia weight is 0.9, the minimum inertia weight is 0.4, and the position of particles is at [0, 1].

## 3. Results Discussions of Modeling

### 3.1. ELM Prediction Model

**Figure 5:**
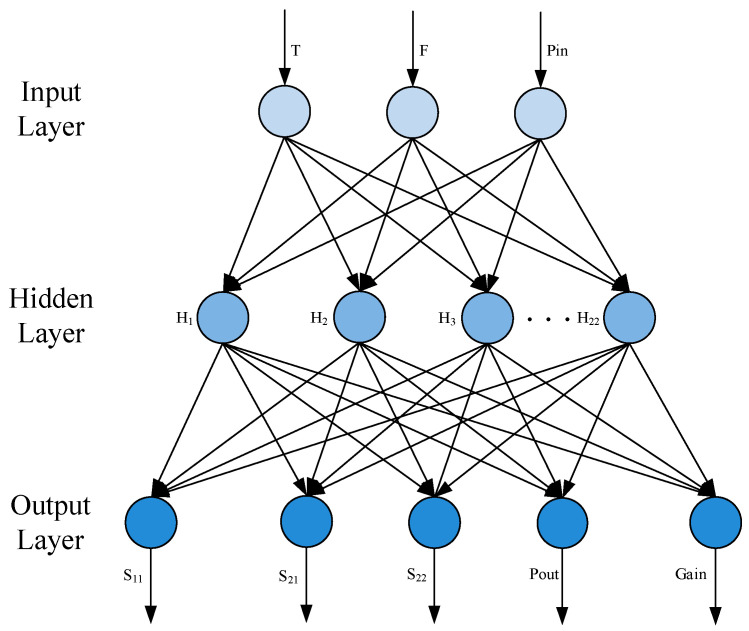
Prediction model based on ELM for GaN PA performance.

Then, the prediction model based on ELM is built as the structure of 3-12-5 as shown in Figure 5. As shown in Table 1, the input vector is composed of the temperature T, frequency F, and the input power Pin. Moreover, the output vector consists of the small signal gain of S_21_, input return loss of S_11_, output return loss of S_22_, output power of Pout, and Gain. Meanwhile, S_11_ is the input return loss, S_22_ is the output return loss, S_21_ is the small signal gain, Pin is the power of the input signal, Pout is the available power of the output two-port network, and Gain is the ratio of output signal power (watt) to input signal power (watt), as shown in Equation (8) [27]:

(8) $$Gain=\frac{Pout}{Pin}$$

In the ideal case, the circuit is expected to output a low S_11_, which indicates that the input ports are matched well. This can lead to smaller losses and facilitate efficient power transfer to the circuit. Therefore, the value of S_11_ directly affects the transmission efficiency of Pin.

Here, T is chosen from 40 °C to 120 °C with a step of 40, F is from 0.2 to 5 GHz with a step of 20 MHz, and Pin is fixed at 29 dBm.

In detail, the input neutron is 3, which can be expressed as the vector of [F, Pin, T], and the output neutron is 5, which is expressed as the vector of [S_11_, S_21_, S_22_, Pout, Gain]. Finally, the neuron number of the hidden layer is determined as 12.

**Figure 6:**
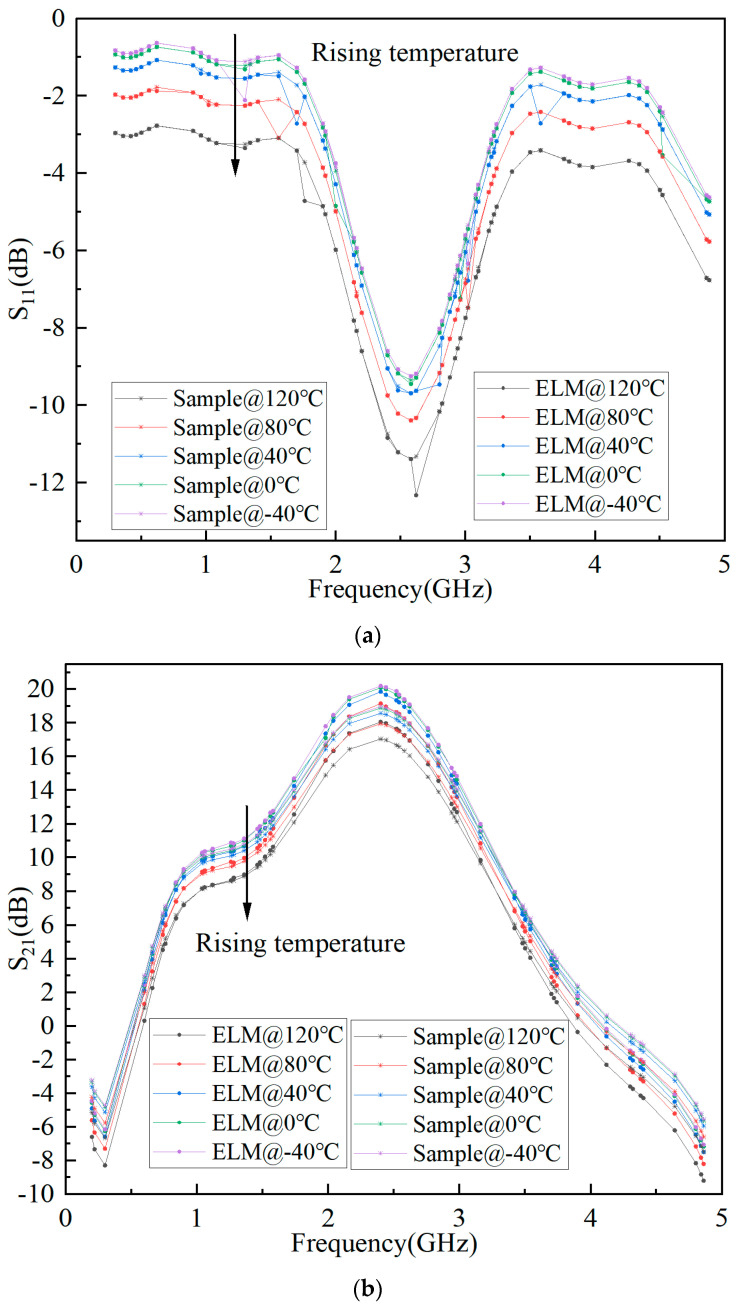

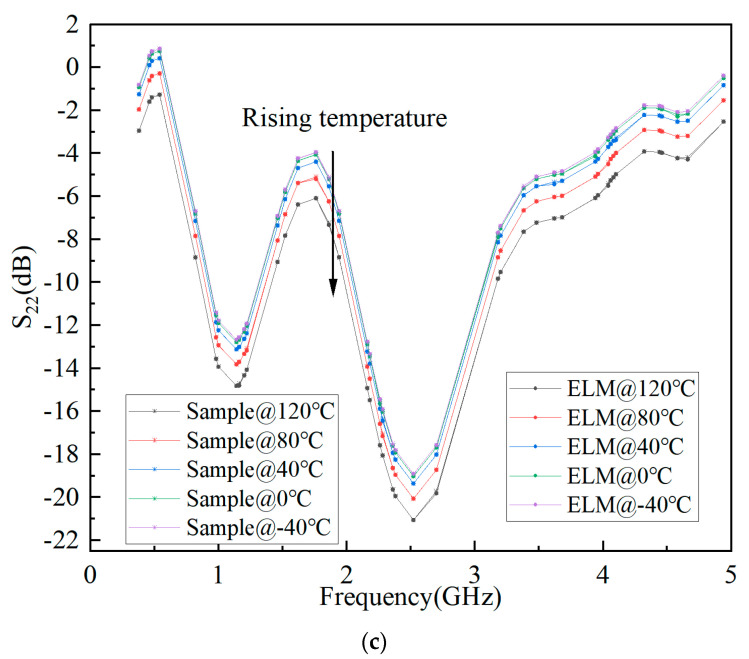
Prediction results of S-parameters based on ELM for GaN PA. (**a**) S_11_. (**b**) S_21_. (**c**) S_22_.

**Figure 7:**
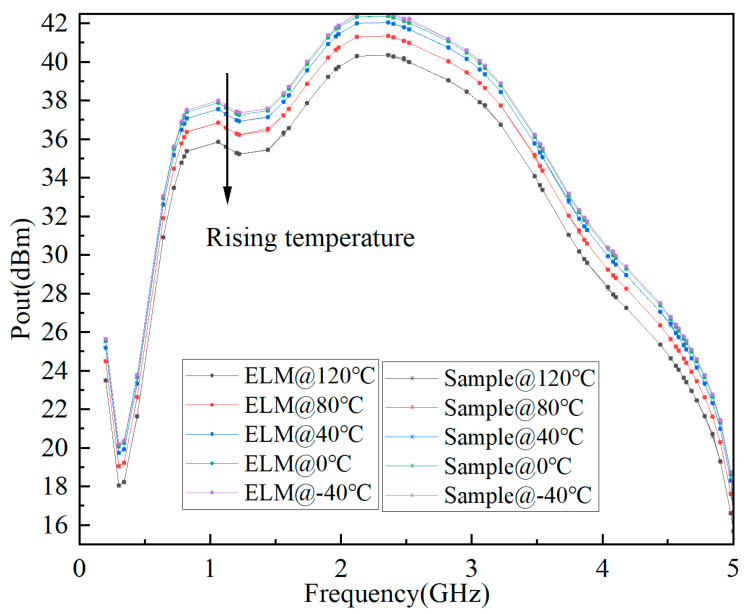
Prediction results of Pout based on ELM for GaN PA.

**Figure 8:**
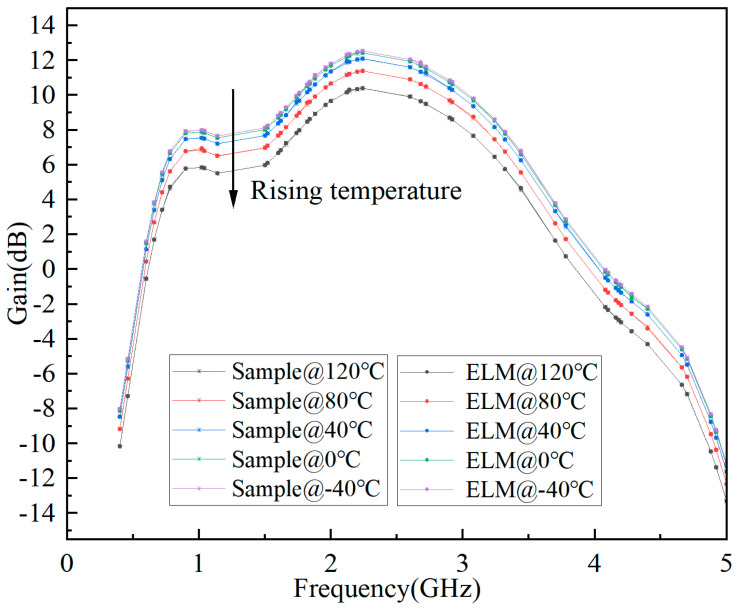
Prediction results of Gain based on ELM for GaN PA.

The predicted results of S-parameter, Pout, and Gain based on ELM are shown in Figure 6, Figure 7, and Figure 8, respectively. It can be seen that the fitting results of S_11_, S_21_, Pout, and Gain are better, and the minimum MSE can reach 0.0109.

### 3.2. PSO-ELM Prediction Model

**Figure 9:**
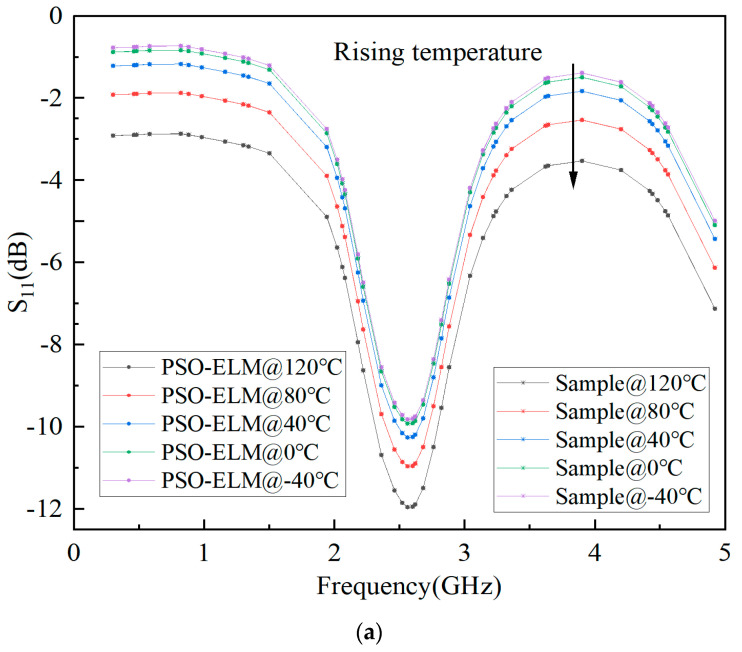

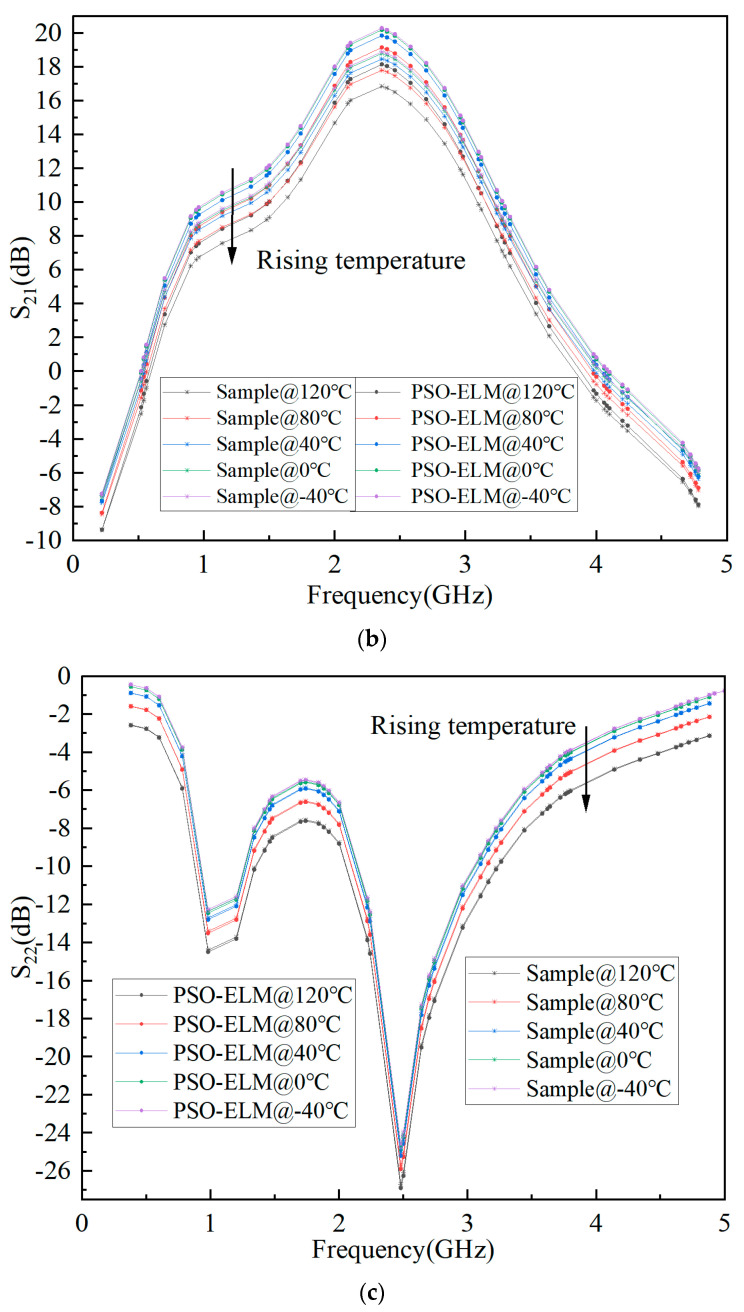
Prediction results of S-parameters based on PSO-ELM for GaN PA. (**a**) S_11_. (**b**) S_21_. (**c**) S_22_.

**Figure 10:**
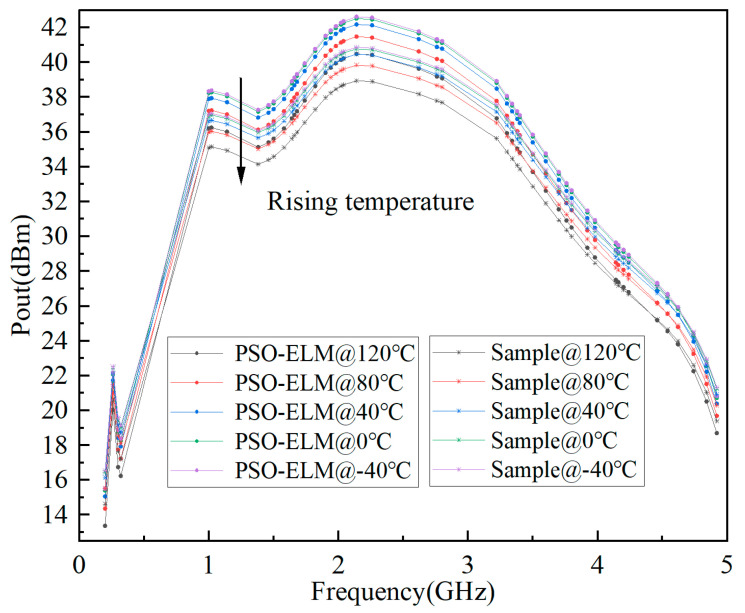
Prediction results of Pout based on PSO-ELM for GaN PA.

**Figure 11:**
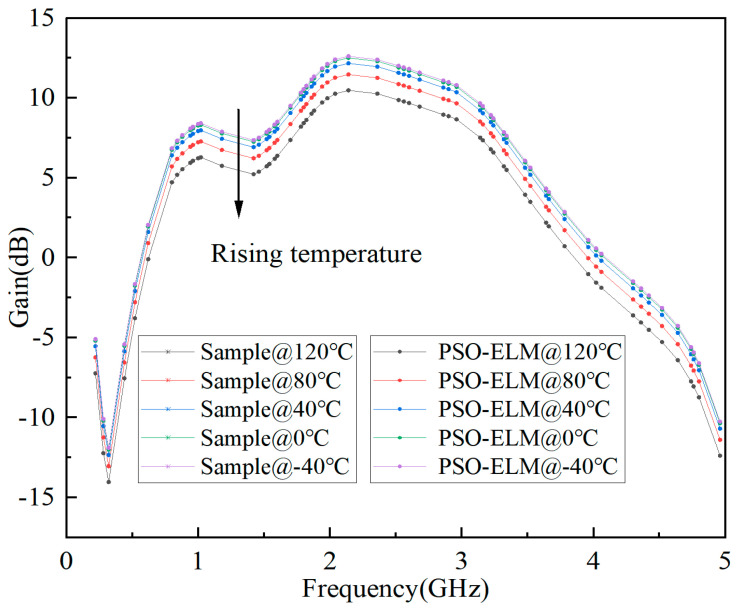
Prediction results of Gain based on PSO-ELM for GaN PA.

The prediction model based on PSO-ELM is the same as ELM, as shown in Figure 5. In order to compare the accuracy of the two models, the same input is used. Finally, the predicted results of the S-parameter, Pout, and Gain based on PSO-ELM are shown in Figure 9, Figure 10 and Figure 11, respectively. It can be seen that the fitting results of S_11_, S_21_, Pout, and Gain are better, and the minimum mean square error can reach 0.0006.

### 3.3. Comparison of Modeling Results between the Two Methods

Moreover, the MSE of ELM and PSO-ELM are given with the same number of neurons in the hidden layer as in Table 2. It can be seen that the accuracy of prediction results based on PSO-ELM is 0.0006 compared to 0.0109 in ELM with 12 neurons of the hidden layer. Meanwhile, it can be seen that with the increased number of neurons in the hidden layer, the prediction accuracy of PSO-ELM is superior, and the prediction accuracy can be improved by increasing the number of neurons in the hidden layer.

Then, the two models in this paper are compared with other methods presented in terms of MSE, as shown in Table 3. From the perspective of training error, the accuracy of the other models is lower than PSO-ELM. It can be concluded that the PSO-ELM has superior advantages of accuracy and handleability, and it is more suitable for modeling large signal behaviors of GaN HEMTs.

## 4. Conclusions

In order to accurately grasp the non-relationship between temperature and PA performance, PSO-ELM and ELM have been used to construct the nonlinear modeling. The prediction results show that the mean square error based on PSO-ELM is 0.0006, and its prediction results and errors are better than ELM. Moreover, it is also compared with other models and it also can present a superior performance. In conclusion, this study is of great significance to optimize the circuit design and improve the reliability of PAs in complex environments.

## Figures and Tables

**Table 1:** Input and output vectors of the models.

Vectors		Range	Step Size
Input Vector	Frequency(F)	0.2 GHz–5 GHz	20 MHz
	Input power (Pin)	0 dBm–30 dBm	0.1 dBm
	Temperature(T)	−40 °C–120 °C	40 °C
Output Vector	Small signal gain (S_21_)	-	-
	Input return loss (S_11_)		
	Output return loss (S_22_)		
	Output power (Pout)		
	Gain		

**Table 2:** Comparison of modeling results.

Model	Number of Neurons in the Hidden Layer	MSE
ELM	2	0.1908
	4	0.1248
	6	0.0800
	8	0.0948
	10	0.0739
	**12**	**0.0109**
	14	0.9903
PSO-ELM	2	0.0720
	4	0.0060
	6	0.0083
	8	0.0090
	10	0.0055
	**12**	**0.0006**
	14	0.0063

**Table 3:** Performance comparison of models.

References	Research Object	Model	MSE
[13]	COMS	SVM	0.0219
[15]	COMS	BPNN	0.0008
[28]	GaN HEMT	ELM	0.0027
**This work**	**GaN HEMT**	**ELM**	**0.0109**
		**PSO-ELM**	**0.0006**

## Data Availability

The original contributions presented in the study are included in the article, further inquiries can be directed to the corresponding author.
